# Anomalous 3D nanoscale photoconduction in hybrid perovskite semiconductors revealed by tomographic atomic force microscopy

**DOI:** 10.1038/s41467-020-17012-y

**Published:** 2020-07-03

**Authors:** Jingfeng Song, Yuanyuan Zhou, Nitin P. Padture, Bryan D. Huey

**Affiliations:** 1 Department of Materials Science and Engineering, University of Connecticut, Storrs, CT 06269 USA; 2 School of Engineering, Brown University, Providence, RI 02912 USA

**Keywords:** Materials science, Nanoscience and technology

## Abstract

While grain boundaries (GBs) in conventional inorganic semiconductors are frequently considered as detrimental for photogenerated carrier transport, their exact role remains obscure for the emerging hybrid perovskite semiconductors. A primary challenge for GB-property investigations is that experimentally they need to be performed at the top surface, which is not only insensitive to depth-dependent inhomogeneities but also could be susceptible to topographic artifacts. Accordingly, we have developed a unique approach based on tomographic atomic force microscopy, achieving a fully-3D, photogenerated carrier transport map at the nanoscale in hybrid perovskites. This reveals GBs serving as highly interconnected conducting channels for carrier transport. We have further discovered the coexistence of two GB types in hybrid perovskites, one exhibiting enhanced carrier mobilities, while the other is insipid. Our approach reveals otherwise inaccessible buried features and previously unresolved conduction pathways, crucial for optimizing hybrid perovskites for various optoelectronic applications including solar cells and photodetectors.

## Abstract (web summary)

The role of grain boundaries (GBs) in halide perovskite is an interesting topic but existing investigations are limited to the top surface. Here Song et al. employ tomographic AFM to study the buried features of grains and GBs, revealing coexistence of interconnected conducting and inert GBs.

## Introduction

Hybrid perovskites (HPs) such as methylammonium lead triiodide (CH_3_NH_3_PbI_3_ or MAPbI_3_) are of tremendous interest due to their superior optoelectronic properties^1–4^ such as high light absorption coefficients, long photogenerated carrier diffusion lengths, and suitable carrier mobilities. In particular, the use of HPs in solar cells has led to high power conversion efficiencies, up to certified 25.2%^5^. Furthermore, HPs allow for low-temperature, high-throughput solution processing, and therefore offer promising, low-cost alternatives to conventional silicon-based systems for solar cells and other optoelectronic device applications^6–8^. Typically, the MAPbI_3_ HP thin films are polycrystalline in nature, with average grain sizes ranging from 100 nm to a few microns^9,10^. This can result in extremely high internal interfacial areas or grain boundaries (GBs), such that it is imperative to fully characterize and either leverage or mitigate the effects of GBs on photogenerated carrier transport in order to optimize future device performance.

However, the impact of GBs on the performance of HP solar cells is still being heavily debated^11–15^. Some studies show that GBs are detrimental with shorter carrier lifetimes due to non-radiative recombination^15^, while others suggest that HP GBs are only moderately detrimental, especially when compared to conventional polycrystalline photovoltaics and optoelectronics^16–19^. Still others report that HP GBs are benign^20,21^, or even beneficial^11^. Recent works additionally revealed that GBs in MAPbI_3_ HPs could exhibit depth-dependent^22,23^ or crystallinity-dependent^24^ electrical properties. However, these studies are primarily based on characterization of the top surface of HPs where the GBs intersect the surfaces^11,13–15,17–20,22,24–26^, which are not only likely to be insensitive to inhomogeneities in composition and microstructure as a function of depth, but also could be susceptible to topographic crosstalk. Cross-sectional scanning electron microscopy (SEM) and transmission electron microscopy (TEM) have revealed sub-surface grain morphologies and elemental distributions, but so far these high-energy, electron-beam-based measurements (e.g. electron-beam induced current mapping) are limited to 2D studies, and also they do not resolve local photoconductive properties due to the absence of light illumination^9,27,28^. In this overall context, direct 3D nanoscale measurements of through-thickness photoconductive behavior in MAPbI_3_ HP thin films are critically needed, from the top surface through to the underlying electrode, for determining the true contribution of GBs to carrier transport.

To that end, we employ tomographic atomic force microscopy (T-AFM) to acquire a fully-3D, nanoscale, photogenerated carrier transport map throughout the thickness of a polycrystalline MAPbI_3_ HP thin film. This approach essentially implements continuous in situ nanomachining by the scanned AFM probe, in order to progressively planarize a specimen site-selectively, and thus to gradually uncover otherwise buried microstructural features of interest. Coupled with sequential or simultaneous property mapping^29–31^, here photoconductive AFM (pc-AFM)^32^, enables the acquisition of tens to hundreds of high-fidelity photocurrent maps over a range of polished depths. In this manner, most MAPbI_3_ GBs are clearly discovered to act as highly interconnected 3D pathways for enhanced photogenerated carrier transport. These GBs exhibit photoconductivity that is up to three times higher than adjacent grains and obey similar drift-driven conduction mechanisms according to voltage-dependent measurements, which collectively suggest a higher effective carrier mobility at GBs. For approximately 5% of morphologically indistinguishable GBs, on the other hand, the photoconduction behavior is essentially insipid throughout the film thickness, with no enhancement in vertical conduction and uninhibited lateral carrier transport. These results confirm the substantial advantage of fully 3D nanoscale property mapping for fundamentally understanding, and ultimately optimizing, charge transport in HP thin films in devices.

## Results

### 3D photocurrent mapping using T-AFM

**Fig. 1:**
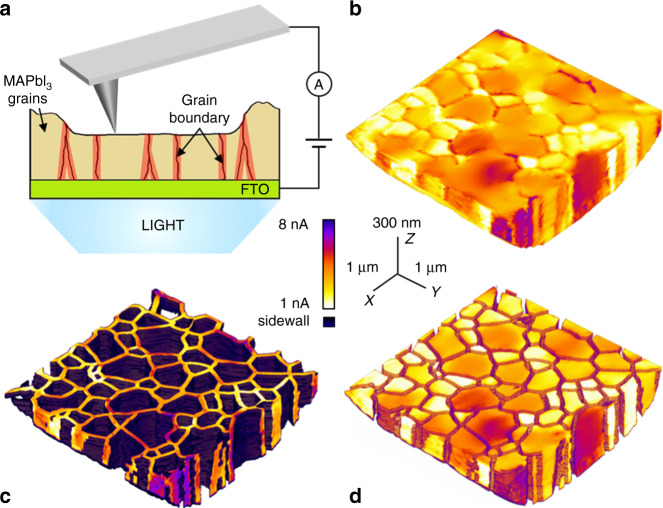
Fully 3D photocurrent mapping throughout polycrystalline MAPbI_3_ thin films. **a** Schematic illustration of photoconductive T-AFM with bottom illumination of polycrystalline MAPbI_3_ thin film. **b** Volumetric perspective of photocurrent distribution throughout MAPbI_3_ thin film by T-AFM. **c**–**d** Volumetric perspectives of photocurrent segmented along 3D GB network (**c**) and 3D grains (**d**). (**b**)–(**d**) share the same scale bars and linear-scale color contrast (center).

We present results from uniform MAPbI_3_ thin films (around 560 nm thick) deposited on fluorine-doped tin-oxide (FTO) coated glass substrates (fabrication details are described in Methods). As shown in Supplementary Fig. 1, this thin-film perovskite is phase-pure and comprises grains with lateral sizes ranging from 100 nm up to almost 2 µm. The 3D photocurrent mapping is based on a back-illuminated, contact-mode AFM platform as illustrated schematically in Fig. 1a, including an integrated current detector and a conducting AFM probe. However, unlike conventional low-force pc-AFM^33–35^, our tomographic approach employs loads as high as µNs to the conductive tip. Accordingly, consecutive T-AFM scans can effectively polish the surface by removing layers as thin as a single unit cell in some cases^31^. Here an average of 16 nm of MAPbI_3_ are milled away with each sequential image frame until the FTO bottom layer is reached; for comparison, this is equivalent to the diagonal nearest neighbor distance between initial image pixels. Within around the first 5 scans, the root-mean-square (RMS) roughness diminishes by more than one order of magnitude from the as-received surface (Supplementary Fig. 2). The excavated material is swept out of the imaging field by the rastering tip, which is apparent in Supplementary Figs. 2 and 3. A cross-sectional schematic illustration, scaled identically in *x* and *z*, is also included in Supplementary Fig. 3, demonstrating that the probe aspect ratio and tip length do not contribute to the photocurrent measurements within the excavated regions.

Since photoconductive AFM is performed simultaneously with such progressive nanomachining, local properties are therefore acquired as a function of depth. To interpret and visualize these results via conventional image analysis, the ensemble of as-acquired data is interpolated into rectilinear voxels to reconstruct the 3D photocurrent distribution throughout the entire scanned volume. The perspective view of Fig. 1b typifies the resulting photocurrent tomogram for the polycrystalline MAPbI_3_, in this case based on 34 initial images which overall comprise 7.6 million total pixels (11.7 nm × 11.7 nm) of photocurrent and (*x*, *y*, *z*) position data. More details of the T-AFM procedure can be found in Methods as well as Supplementary Fig. 4, including a discussion of physical damage. Analyzing the milling and interpolation distances reveals more than 95% of all tomogram voxels are within just two adjacent nearest neighbors from the initially acquired data. Throughout, photocurrents ranging from 100 pA to 10 nA are consistently measured, while only less than 10 pA dark current is detected under the same DC bias (Supplementary Fig. 5).

Segmenting the data according to local photocurrent magnitudes enables easy visualization and analysis of grains and GBs separately, as shown in Fig. 1c, d, respectively. Clearly, the GBs in polycrystalline MAPbI_3_ films form highly interconnected 3D conductive channels extending from the top surface down to the bottom FTO layer, most oriented nearly perpendicular to the substrate. Moreover, according to the 3D and consecutive cross-sectional views (see Supplementary Movies 1–3), the photocurrent measured from MAPbI_3_ GBs are consistently higher than the ajacent grains, independent of the remaining film thickness, grain size, or grain shape.

### Nanomachining of MAPbI_3_ thin film using T-AFM

**Fig. 2:**
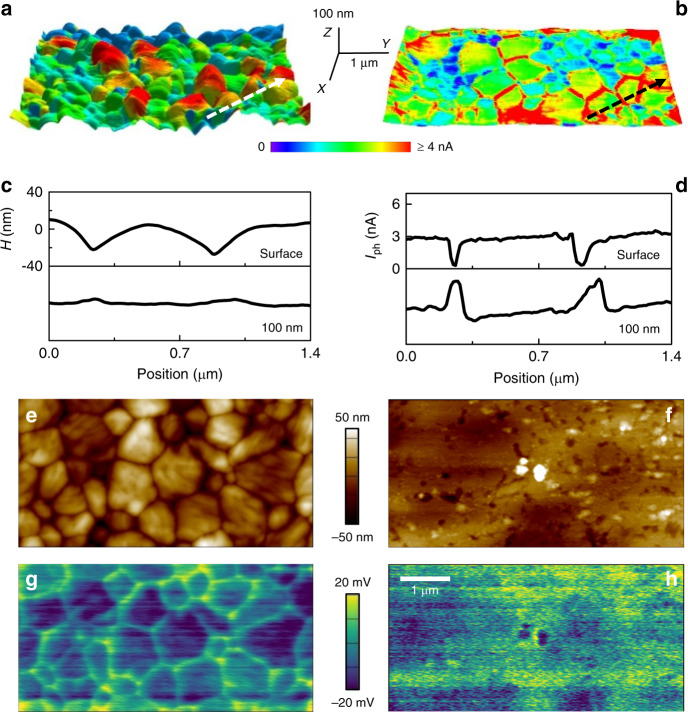
Nanomachining of MAPbI_3_ perovskite thin film using T-AFM. **a**–**b** 3D perspective of MAPbI_3_ thin-film topography overlaid by photocurrent color contrast for a single 3 µm × 6 µm region, both at the as-received top surface (**a**) and after nanomachining 100 nm into the film (**b**). **c** Topography line-scans indicated by arrows in (**a**, **b**). **d** The simultaneously acquired corresponding photocurrent line scans which evidence relative signal inversion beneath the as-received surface. **e**–**f** Similar topography images pre- and post-nanomachining for the same sample, with matching **g**, **h** surface potential maps also indicating dramatic contrast variations at and beneath the surface. Images (**e**)–(**g**) share the same scale bar with (**h**).

Beyond revealing sub-surface 3D conduction network at GBs, it is noteworthy that the effective site-selective nanomachining during T-AFM likely improves image resolution and confidence compared to the initial surface. This is due to the substantially smoothened surface topography, effectively minimizing common AFM imaging artifacts. Examples include tip–sample geometric convolution, as well as apparent but unreal property correlations with local changes in slope or curvature resulting from measurement sensitivity to the tip-sample contact area^36^. Figure 2a–b depicts representative photocurrent maps superimposed on identically scaled surface topographies before (a) and after (b) the T-AFM nanomachining. In addition to a significantly smoother surface morphology as demonstrated by line scans (Fig. 2c, where indicated in a–b), there is also a complete inversion of the relative photocurrent magnitude (Fig. 2d). Corresponding plan-view images further demonstrating this relative photocurrent inversion are displayed in Supplementary Fig. 6. Specifically, while the photocurrent in the grains remains stable at 3 nA, the intervening GB is initially less photoconductive (only 0.3 nA) but it increases 20-fold to 6 nA at 100 nm beneath the surface (and as far down as 430 nm, Supplementary Fig. 7). Kelvin probe force microscopy (KPFM) measurement during illumination, another method frequently used to assess HPs, exhibits a similar tendency. For the relatively rough as-grown MAPbI_3_ thin-film surface (Fig. 2e), we record higher surface potentials at GBs (Fig. 2g) compared to adjacent grains. When the surface is polished to the nanoscale (Fig. 2f), the GB potential contrast is eliminated (Fig. 2h). Previous studies have observed grain orientation and facet-dependent responses on as-received surfaces^37^, while in our case, surface reconstructions and facets are physically removed by the nanomachining process (Fig. 2f and Supplementary Fig. 2). It is therefore unsurprising, and could generally be advantageous, that these possibly spurious contributions to surface potential or photocurrent measurements become negligible^38,39^.

MAPbI_3_ is furthermore known to be sensitive to ambient exposure, and also specimen processing for any given sample may lead to intended or unintended composition variations near the surface^8^. According to our T-AFM results, collectively these circumstances likely explain much of the disagreement in the literature about enhanced, reduced, or equal photocurrents as well as surface potentials for GBs or individual grains^11,20,22,25^. Here, such discrepancies can result from unidentified and often uncontrollable variations in as-received surface properties and topography-related artifacts, for surface geometries similar to those encountered elsewhere^11,20,27^. Upon nanomachining during T-AFM, such effects are sufficiently alleviated that future investigations could directly probe ingress into the depth of HP thin films by beneficial dopants, defect-passivating species, migrating ions, and degradation-inducing environmental species, or reaction products such as acetic acid from the breakdown of common solar cell packaging materials over time^40^.

### *I*_ph_–*V* behavior of grains and GBs

**Fig. 3:**
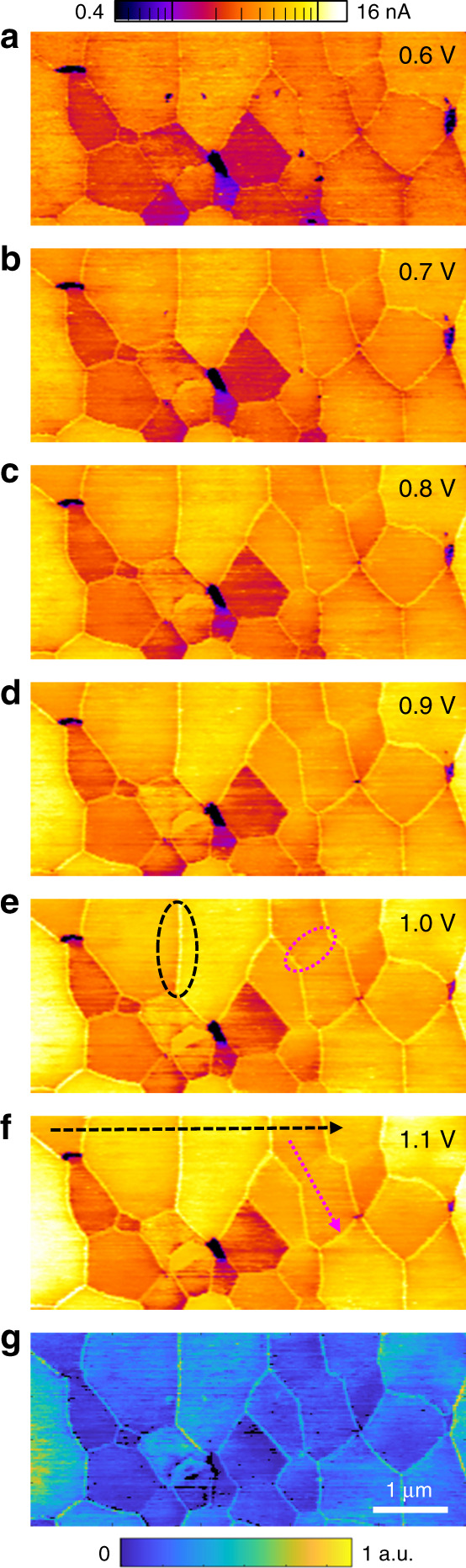
2D mapping of photocurrent-voltage characteristics. **a**–**f** Montage of pc-AFM maps in a single 3 µm × 6 µm T-AFM polished region for sequentially increasing biases from 0.6 V (**a**) up to 1.1 V (**f**). **g** 2D map of the calculated local product of *µN*_ph_, which incorporates the slopes from pixel-by-pixel linear fitting of the photocurrent *vs*. bias from (**a**) to (**f**) according to the drift equation. All of (**a**)–(**f**) share the same log-scale color contrast (top).

Once surface artifacts and inhomogeneities are literally swept away, high-fidelity 2D mapping of the photocurrent–voltage (*I*_ph_–*V*) characteristics of polycrystalline MAPbI_3_ thin films becomes practical at any depth of interest. This is based on series of low-load and hence non-milling pc-AFM images on a T-AFM polished region, each with a distinct potential difference between tip and FTO back electrode. The montage of 3 µm × 6 µm photocurrent maps in Fig. 3a–f, approximately 100 nm below the initial surface, indicate that GB photocurrents are uniformly enhanced upon biasing just as with the depth-dependent results of Figs. 1, 2.

We analyze these results according to the photocurrent drift equation of *I*_ph_ = *qN*_ph_*µAV*/*L*, where *q* is the unit charge, *N*_ph_ is an unknown photogenerated carrier density, *µ* is the unknown but crucial charge carrier mobility, *A* is the tip-sample contact area, *V* is the applied voltage, and *L* is the MAPbI_3_ film thickness. Uniquely, since our pc-AFM measurement is conducted on a significantly polished flat surface by T-AFM, the otherwise locally varying *L* and *A* terms can be treated as constant during the voltage dependent scans. Therefore, it is possible to estimate in a pixel-by-pixel fashion the technologically critical product of *µN*_ph_ from the slopes of the linearly fitted *I*_ph_–*V* curves, as displayed in Fig. 3g. It reveals a range of possible *µN*_ph_ products, which are essentially consistent across any given grain. Enhancements of carrier *µN*_ph_ products at GBs, too, appear to be decoupled from the adjacent grain morphology. Practically, the calculated Fig. 3g encompasses around 32,000 distinct *I*_ph_–*V* spectra, each based on photocurrent acquired with applied voltages from 0.6 to 1.1 V.

As the MAPbI_3_ thin films investigated herein are directly prepared on FTO, there is no appreciable built-in field as with a fully assembled pn-junction solar cell. Therefore, as is shown in Supplementary Fig. 8, pc-AFM measurements on T-AFM nanomachined regions at voltages approaching short-circuit conditions (0–0.3 V) yield no clear image of distinguishable grains and GBs. Similarly, high voltages beyond 1.2 V may degenerate the conductive coating on the probe, while negative biasing either results in poor fidelity images at low bias or damage to the sample surface at higher biases beyond −0.4 V (Supplementary Fig. 9). Therefore, in the interest of consistency, Fig. 3 is based only on the high signal-to-noise ratio photocurrent maps acquired with DC biasing from 0.6 to 1.1 V. T-AFM investigations of the voltage and tomographic performance of fully assembled cells, near short-circuit as well as open-circuit conditions, is a promising but necessarily future endeavor. Regardless, the consistently higher photocurrents measured at GBs, different photocurrents for distinct grains, and also variations within grains and possibly along individual grain boundaries, are all compatible with spatial variations reported by others for MAPbI_3_ thin films^13,14,22,24,25,41,42^.

Statistically, for any given set of grains and grain boundaries in a field of view, there are often one or more that exhibit particularly strong (or weak) photocurrents or calculated carrier *µN*_ph_ products. These appear in arbitrary locations as noticed when comparing Fig. 3g and Supplementary Figs. 8 and 9. Further data analysis on the lateral, vertical and grain-to-grain variations of photocurrents are presented in Supplementary Figs. 10–12. And while much higher voltage-resolution sweeps have been reported with pc-AFM “parked” at single or arrayed locations on MAPbI_3_ thin films^22,34^, the necessary time for both better voltage- and nanoscale-resolved spectra is experimentally impractical, justifying our relatively coarse voltage steps. This is especially reasonable given the theoretical and experimentally measured signal linearity with bias, along with the exceptional signal to noise in the voltage-dependent images afforded by the minimization of surface artifacts during T-AFM.

In fact, since the film thickness available for exciton formation is from constant to around 1% after nanomachining, the exponentially decaying [growing] optical transmission [absorption] generates a knowable local photogenerated carrier density of *N*_ph_ = *ητI*_0_(1 − exp(−*αL*))/*L*, where *η* is the quantum efficiency, *τ* is the carrier lifetime, *I*_0_ is the incident photon flux, and the absorption coefficient *α* is around 6 × 10^4^ cm^−1^ from previous reports^43,44^. *I*_0_ is assumed to be uniform beneath the AFM probe, since the entire field of view is uniformly illuminated. In addition, according to the previous studies on quantum efficiency of individual grains of polycrystalline MAPbI_3_ thin films^27^, and the orientation effect to the optoelectronic properties of MAPbI_3_^45^, the terms *α*, *η*, and *τ* are not significantly dependent on grain size or possibly varying orientations of MAPbI_3_. Therefore, Fig. 3g may be interpreted as a nanoscale-resolved map of the relative photogenerated carrier mobility for our thin-film HP, as to first order *N*_ph_ can be considered as constant. Accordingly, Fig.3 implies higher mobilities at the identified GBs, suggesting that such GBs can act to enhance charge carrier transport and hence be engineered to optimize future device performance.

### 3D identification of two GB types for MAPbI_3_ thin films

Through thorough analysis of the 3D T-AFM and 2D pc-AFM results in Figs. 1, 3, we have identified two GB types for MAPbI_3_ thin films, denoted as Type I and Type II for the analysis and discussions to follow. With Type I GBs, they act as preferred channels for conduction through the film; evidently the direction depends on the nature of the carrier and the field polarity. This would preempt lateral inter-grain carrier diffusion in a normal electric field, also inhibit it upon application of in-plane electric fields, with carriers instead preferring the higher conductivity and path-redundant network of GBs. Such behavior enables the sometimes drastically different photocurrent behavior observed in pc-AFM for adjacent grains, and at GBs, over length scales as small as 10–100 nm. With Type II GBs, on the other hand, vertical conduction is unenhanced and lateral carrier transport is uninhibited. Although there are many published examples of different GB conduction properties for HPs, like those we have directly mapped, theoretical calculations have predicted such intrinsically benign GBs for MAPbI_3_ as well^18,46^. These could result in single-crystal-like behavior for a hypothetical polycrystalline HP exclusively comprising such interfaces^18^, though experimental observation remains scarce for such essentially-perfectly-passivated GBs^21^. So far, most work identifies what we denote as “Type I” GBs, like the interface highlighted by the black dashed oval in Fig. 3e. By minimizing surface artifacts, however, our investigation is also able to reveal another type of boundary that behaves practically identical to the adjacent grains. Highlighted within an overlain magenta dotted oval in Fig. 3e, this “Type II” interface is nearly unrecognizable in the voltage-dependent photocurrent or effective carrier mobility maps.

**Fig. 4:**
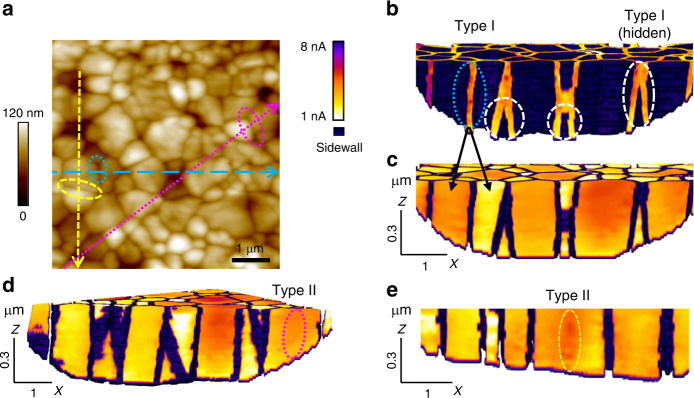
3D identification of two GB types for polycrystalline MAPbI_3_. **a** 6 µm × 6 µm AFM topography of as-grown MAPbI_3_ thin-film surface before T-AFM. **b**–**c** Segmented cross-sectional tomograms of GBs and grains respectively, revealing Type I GBs at and hidden beneath the surface along the blue long-dashed line from (**a**). **d**–**e** Cross-sectional tomogram of Type I as well as insipid Type II GBs, along profiles respectively indicated by magenta dotted line and yellow dashed line in (**a**). Specific interfaces highlighted by overlain ovals in (**a**) are equivalently identified in (**b**, **d**, **e**), demonstrating absolute through-thickness differences in photocurrents for Type I and II GBs despite being topographically indistinguishable at the surface.

Upon careful correlation between the topography for the as-received film surfaces in Fig. 4a, and the corresponding underlying photoconduction maps uniquely available from our tomographic approach, both of these GB types also become convincingly identifiable. For instance, Fig. 4b, c display cross-sections of tomograms (respectively from Fig. 1c, d), segmented to separate the 3D GB network and the 3D grain photoconductivity. Multiple Type I GBs are obvious, even including several which would never be detected with conventional surface measurements because they only exist deep within the film (white dashed overlays in Fig. 4b). In every case, especially notable on the left side of Fig. 4b (blue dotted oval), higher and lower photocurrents within the two neighboring grains (indicated by black arrows in Fig. 4c) clearly exhibit the proposed Type I behavior which preempts lateral inter-grain carrier diffusion. For the much more difficult to find Type II interfaces at the surfaces, on the other hand, they become easy to uncover within T-AFM results-as demonstrated by Fig. 4d and e where indicated from Fig. 4a. In each case, two apparently distinct grains are separated by a topographic grain boundary groove that is morphologically indistinguishable from any other GBs (Supplementary Fig. 13), while the photocurrent at and beneath the surface remains uniform and unremarkable. Notably, neither Type I or Type II GBs are at all correlated to low or high load AFM scan directions, in these specific instances orientated by −85° (blue oval, but also numerous other Type I examples), −20° (yellow oval, Type II) and −70° (magenta oval, Type II) with respect to the horizontally scanning probe (0°).

**Fig. 5:**
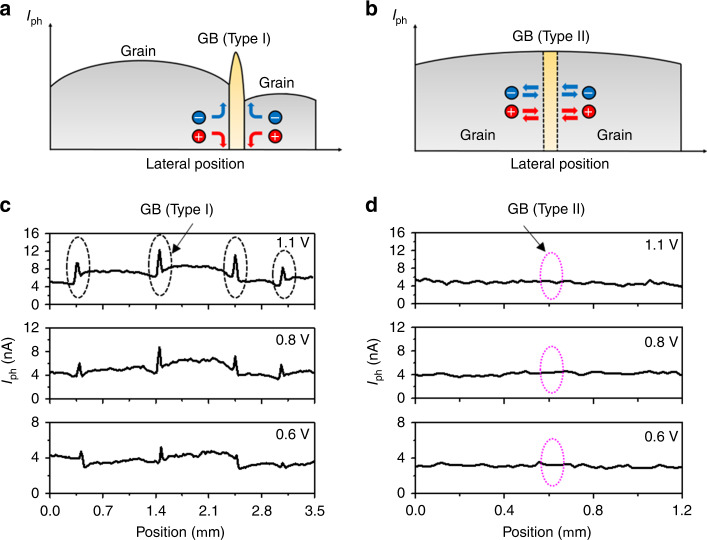
Two GB types revealed in polycrystalline MAPbI_3_ thin film. **a**–**b** Proposed models of Type I and Type II GBs based on the pc-AFM and T-AFM results. **c**–**d** pc-AFM line scans during distinct tip-FTO biasing as noted across Type I (**c**) and Type II (**d**) GBs, revealing voltage-independent unique behavior. The two types of GBs are highlighted with black dashed oval and magenta dotted oval, respectively, here and in Fig. 3e, taken across the paths equivalently identified in Fig. 3f.

We have summarized our proposed models for the two GB types in Fig. 5a, b respectively. Line scans of each type, along the paths identified by the dashed and dotted arrows in Fig. 3f, confirm that the unique character of Type I and Type II GBs persists throughout the probed voltage range (up to 1.1 V, Fig. 5c, d respectively). Together, these depth- and voltage-dependent observations from Figs. 4–5 further confirm that Type I GBs in MAPbI_3_ thin films are enhanced conduction channels, while Type II interfaces are insipid. We estimate an up to 5% density of Type II GBs for our films. It is tempting to attribute the Type II GBs to twin boundaries based on previous theoretical predictions of completely benign GBs^18^ and twin boundaries^46^ in halide perovskites, as well as similar reports in other semiconductor systems such as GaAs^47^. Recent experimental observations on ferroelastic twin boundaries^48^ may provide additional support for this assumption. Additionally, other planar defects such as Ruddlesden-Popper faults could also have similar behavior as Type II GBs. Still, future multi-modal investigations combining T-AFM, pc-AFM, atomic-resolution electron microscopy (SEM, TEM), and advanced optical or x-ray methods, may ultimately be needed to thoroughly resolve the microstructure and character of the Type II GBs.

## Discussion

Based on the unique T-AFM approach, we have successfully demonstrated a fully-3D nanoscale map of photogenerated carrier transport throughout MAPbI_3_ HP thin-film grains and GBs. We clearly show that a network of Type I GBs provides 3D interconnected charge carrier pathways. We also discover the coexistence of up to 5% of effectively benign Type II interfaces with unenhanced vertical conduction and uninhibited lateral carrier transport. To first order, a higher carrier mobility is implicated for Type I boundaries, and not observed whatsoever for Type II GBs. These insightful results confirm the clear advantage of tomographic studies such as T-AFM, transcending the limitations of conventional surface-based characterizations of HPs and related materials systems. Such work can have profound implications for the future design of HP devices based on grain boundary engineering.

Finally, while this study has a particular focus on revealing intrinsic photogenerated carrier transport in HP semiconductor thin films, by adopting more complex device structures that include carrier-selective layers, it will also become possible to directly investigate 3D carrier transport in configurations and operating conditions that mimic fully assembled optoelectronic devices. Already, T-AFM has uncovered distinctive Type I and Type II GBs. These may be differentially leveraged for future devices, although, there is no single answer as to which GB type is better or preferred. With lateral devices, for instance, strong scattering of charge carriers at GBs can drastically limit the charge carrier diffusion, as reported previously for lateral polycrystalline perovskite solar cells, leading to low PCE of less than 0.1%^49,50^. In such a configuration, a high proportion of Type II GBs instead of Type I would be essential, as such a single-crystal-like system should enhance the optoelectronic performance^18^. For vertical devices such as photodetectors or typically configured solar cells, on the other hand, the device performance will be dominated by vertical charge carrier transport^51^, in which case a preponderance of Type I GBs could be beneficial due to their higher mobility for charge carrier transport. Furthermore, it is enticing to consider that the various GB types may even be chemically tailored^52^ to further enhance their differences and hence the ultimate performance of various HP optoelectronic devices.

## Methods

### Thin-film preparation

The perovskite precursor solution was prepared by co-dissolving 0.159 g MAI (GreatCell, Australia), 0.461 g PbI_2_ (99.9985%, Alfa-Aesar, USA) and 0.014 g MACl additive (GreatCell, Australia) in 0.930 g mixed solvent (DMF/DMSO, 5/1 v/v). The solution was then spin-coated on the substrate at 4500 rpm for 20 s total. At the 10th second after the start of the spin-coating process, 100 mL of toluene antisolvent was dripped in the center of the film. The resulting film was then annealed at 120 °C for 15 min in a dimethylsulfoxide (DMSO) vapor atmosphere. The MACl additive, which volatilized during the annealing process, is for increasing the grain size of the MAPbI_3_ thin films^53^. All steps in the thin-film preparation were performed in a nitrogen-filled glovebox.

### Thin-film characterization

XRD diffraction patterns of the MAPbI_3_ HP thin films were collected using an X-ray diffractometer (D8 Discover, Bruker, Germany) with Cu K*α* radiation (*λ* = 1.5406 Å) at step size of 0.02°. The surface morphology and microstructure of samples were observed using SEM (G500, Zeiss, Germany).

### Photoconductive and Tomographic AFM

All T-AFM and pc-AFM measurements were performed in ambient conditions with an MFP-3D IO-AFM. The KPFM measurement (Fig. 2e–h) was performed under a nitrogen overpressure in an Asylum Research Cypher system. Equivalent KPFM results have also been obtained with the MFP-3D IO-AFM system in ambient. All of our studies used vacuum sealed fresh samples stored in dark and dry conditions. On the timescale of our studies, typically less than 2 h for T-AFM or pc-AFM, there is no obvious evolution of the surface morphology, nor photoresponse decay due to reactions of MAPbI_3_ with the ambient lab environment. This is possibly aided by the nanomachining process, which may remove any surface defects either initially present or which could develop over time. The thin-film sample was obliquely top-illuminated by a 650 nm diode laser (when using the Asylum Research Cypher system), and for most of the work was back-illuminated by a broad-band LED source, providing less than 1 equivalent sun in the visible spectrum of uniform intensity across the imaged field of view (with the Asylum Research IO-AFM). Photocurrent measurements were achieved with an Asylum Research ORCA cantilever holder, with a current resolution of 1 pA and upper detection limit of 20 nA. Ionic currents are considered to be negligible based on the short (approximately 1 ms) pixel acquisition rate with respect to the MAPbI_3_ ion diffusion coefficient (around 10 cm^2^ s^−1^, or 0.1 nm^2^ ms^−1^)^54,55^. This is also supported by the fact that we do not detect any depletion or enhancement of ionic carriers for progressive depths through the film, differing scan rates, or localized measurements within our broader field of view. Without a DC bias applied between the grounded probe and the underlying FTO, photocurrent images are featureless since there is no appreciable built-in field as with a fully assembled pn-junction solar cell. Measurements in dark conditions are also featureless (Supplemantary Fig. 5).

For highest resolution pc-AFM maps (Fig. 3), nN scale forces are applied with Ti/Ir coated probes that nominally exhibit a 25 nm radius of curvature, 75 kHz resonant frequency, and 2.8 N m^−1^ spring constant. For KPFM imaging (Fig. 2g, h), non-contact AC Nap mode imaging with a delta height as low as 5 nm is performed with similar Ti/Ir coated probes. In each of these low-load cases, the topography is unimpacted during progressive images.

For depth-dependent tomographic investigations that effectively polish the surface at the nanoscale with the tip, we employed conductive diamond probes with a nominal 100 nm radius of curvature, 300 kHz resonant frequency, and 50  N m^−1^ spring constant. For a deflection setpoint on the order of 50 nm as implemented, the nominal repulsive force during T-AFM is thus around 2.5 µN. Typical scan rates are 2 Hz, with normal contact-mode settings. This procedure obviously removes material and hence technically “damages” the surface. Aside from changes in depth, however, the positions and signal magnitudes are absolutely consistent for sequential image frames, and in fact improved from the original surface, suggesting that sub-surface damage is negligible during T-AFM. This has already been directly proven via post-TEM cross-sections for functional oxides as well as CdTe photovoltaics^31,32^.

The tomogram in Fig. 1 is based on 34 consecutive pc-AFM scans over a 6 µm × 6 µm region, with nascent pixel sizes of 11.7 nm × 11.7 nm. According to the full width half maximum of GB features in the photocurrent maps, a local lateral resolution on the order of 25 nm is maintained throughout the tomographic imaging process. In the *z*-dimension, the mean resolution is around 16 nm based on the mean local material removal rate throughout the approximately 560 nm thick MAPbI_3_ film. Since removal is not intrinsically constant, however, the raw data is interpolated to rectilinear voxels for further analysis. Even so, as discussed in Supplementary Fig. 4, fewer than 30% of interpolation distances are beyond the separation between a single adjacent nearest-neighbor voxel (11.7 nm), and less than 5% are interpolated beyond 2 adjacent nearest-neighbors. Putting this into perspective, the positions for only around 3% of all tomogram voxels need to be interpolated beyond the FWHM-defined lateral resolution of 25 nm.

We have repeated the voltage and tomographic measurements numerous times with equivalent results.

## Supplementary information

Supplementary Information

Description of Additional Supplementary Files

Supplementary Movie 1

Supplementary Movie 2

Supplementary Movie 3

## Data Availability

The authors declare that the data related to this study are available upon reasonable request.
