# Supplementary material for: Anomalous 3D nanoscale photoconduction in hybrid perovskite semiconductors revealed by tomographic atomic force microscopy
Source: Nat Commun. 2020 Jul 3;11:3308. doi: 10.1038/s41467-020-17012-y (PMC7335063; doi:10.1038/s41467-020-17012-y)
Supplement: Supplementary file 1 — Supplementary Information [file 41467_2020_17012_MOESM1_ESM.pdf]

Supplementary Information

Anomalous 3D Nanoscale Photoconduction in Hybrid Perovskite Semiconductors Revealed by Tomographic Atomic Force Microscopy

Song et al.

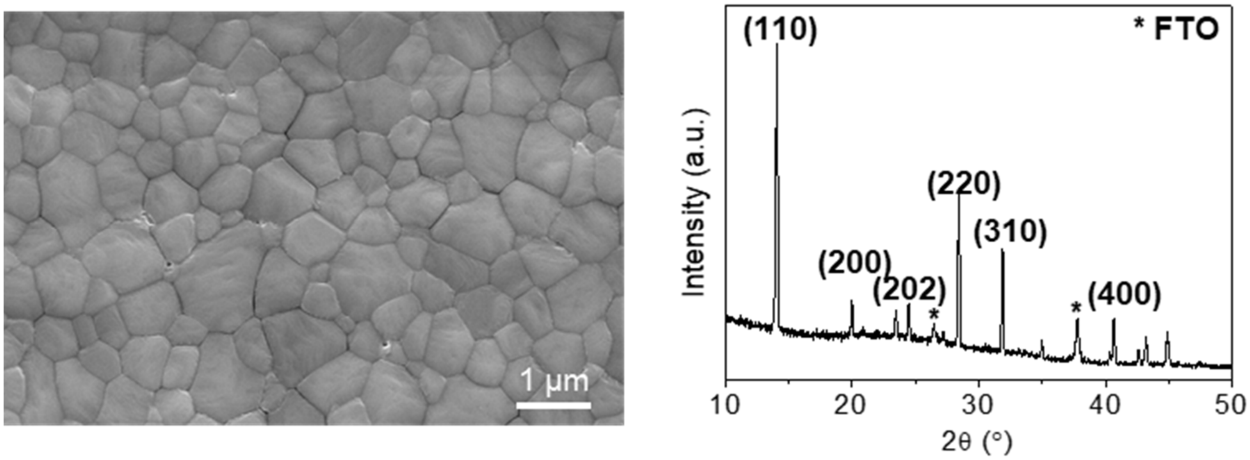

Supplementary Figure 1 | Characterization of the $MAPbI_3$ thin films. SEM and XRD results of the $MAPbI_3$ polycrystalline thin films. The $MAPbI_3$ thin films exhibit a uniform surface with grain sizes ranging from 100 nm up to 1.5 µm, consistent with the AFM topography measurement on an otherwise identical specimen. X-ray diffraction results confirm that the $MAPbI_3$ thin film is polycrystalline and phase pure.

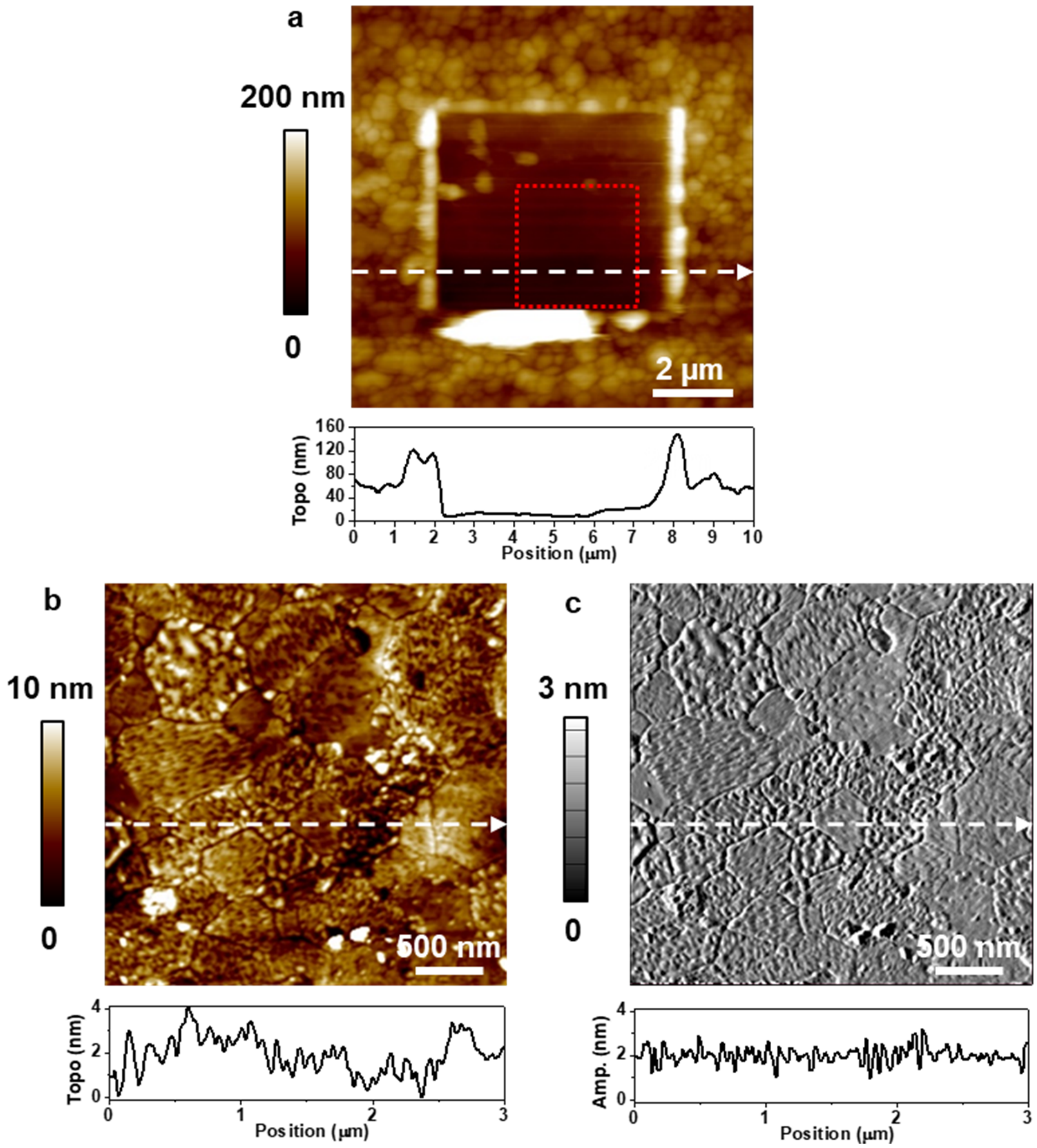

Supplementary Figure 2 | AFM topography of the nanomachined surface. a, Low magnification AFM topography of a $MAPbI_3$ thin film following nanomachining of a square region, milling approximately 50 nm deep into the film and sweeping the removed material to the scanned periphery. **b-c**, AC-mode AFM topography and amplitude (edge identifying) images zoomed-in to the dotted region confirming substantial smoothing following milling. Line profiles along the dashed arrows for each image are also shown.

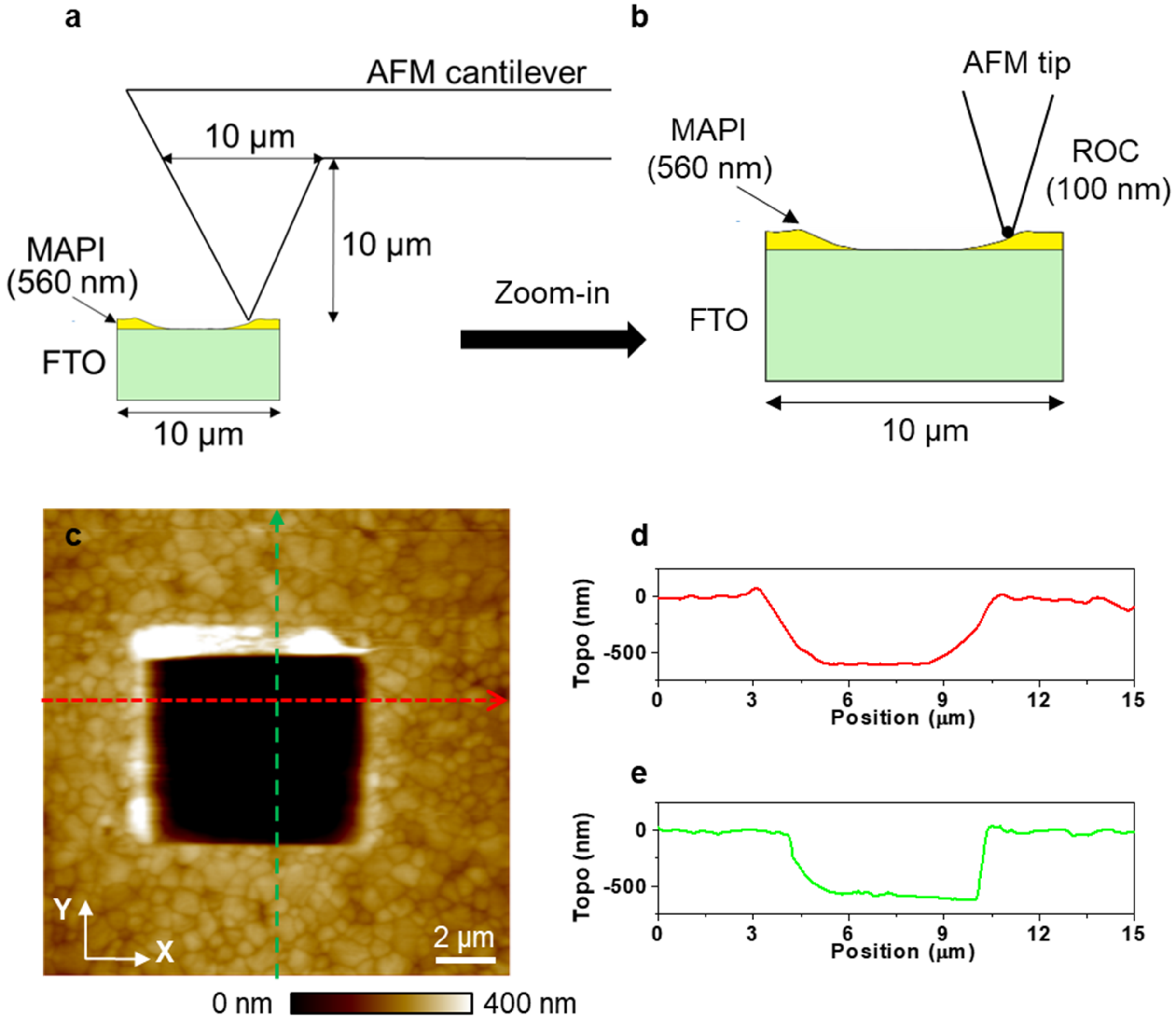

Supplementary Figure 3 | AFM cantilever schematics and post T-AFM surface topography. a-b, The cross-sectional schematic of the AFM cantilever and the $MAPbI_3$ thin film sample during T-AFM process. The magnified view shows a more precise geometrical matching of the AFM tip apex and $MAPbI_3$ thin film thickness. Note the cone angle of the diamond coated tip apex region (around 28°) is smaller than the overall cone angle of the whole AFM tip body part showing in the overall view on the left. **c-e,** Surface topography and line scan profiles (corresponding to the red and green dashed lines on (**c**) respectively) after through-thickness T-AFM.

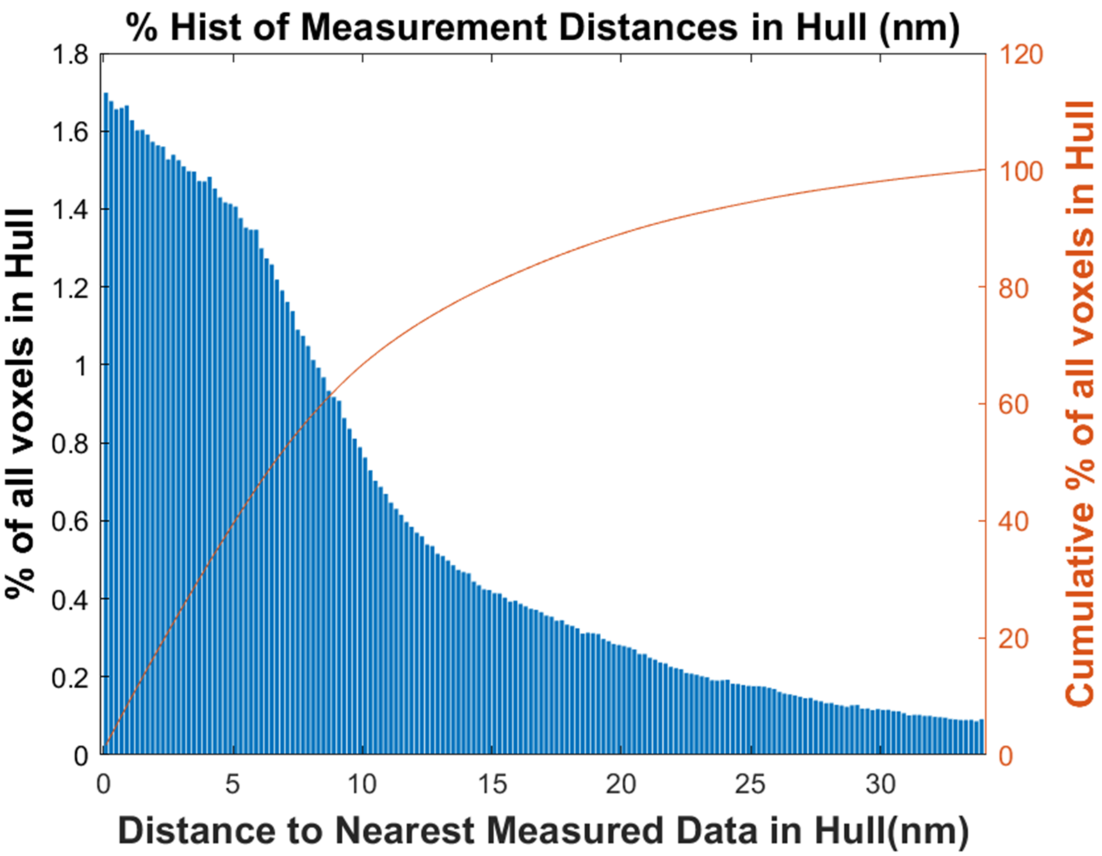

Supplementary Figure 4 | Interpolation distances and overall statistics between sparsely acquired pixels and gridded final tomograms. Histogram of the percentage of tomogram voxels within the milled 'hull' (bar graph, left axis), along with a Pareto sum of these statistics (line plot, right), demonstrating that the position for more than 70% of tomogram voxels are interpolated to within 1 adjacent nearest neighbor initial pixel position (11.7 nm each), more than 95% are within 2 adjacent nearest neighbors, and 99% are within 2 diagonal nearest neighbors (33.1 nm).

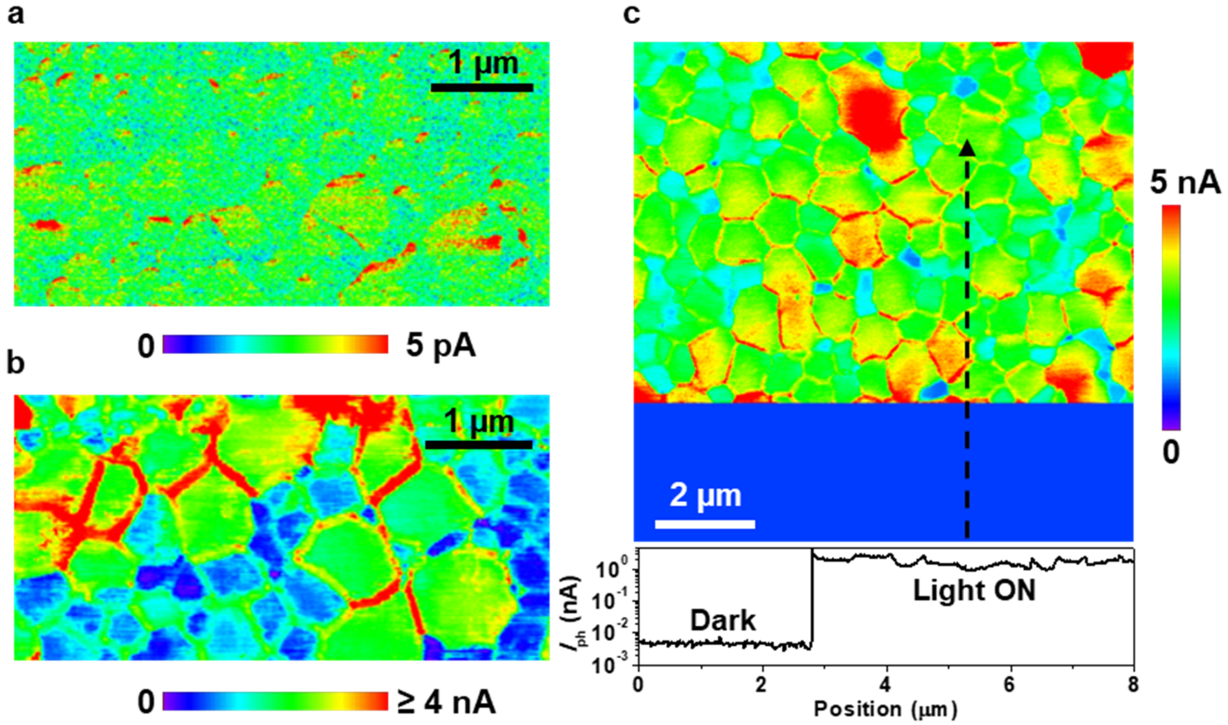

Supplementary Figure 5 | Dark and light current measurement during T-AFM. a-b, Photocurrent mapping of the same region as in Figure 2 without (**a**) and with (**b**) illumination. **c**, Photocurrent mapping of a similar area upon switching the illumination source from OFF (Dark, base) to ON (top). The corresponding line scan (log scale) along the indicated arrow confirms an increase in the mapped current by 3 orders of magnitude upon illumination.

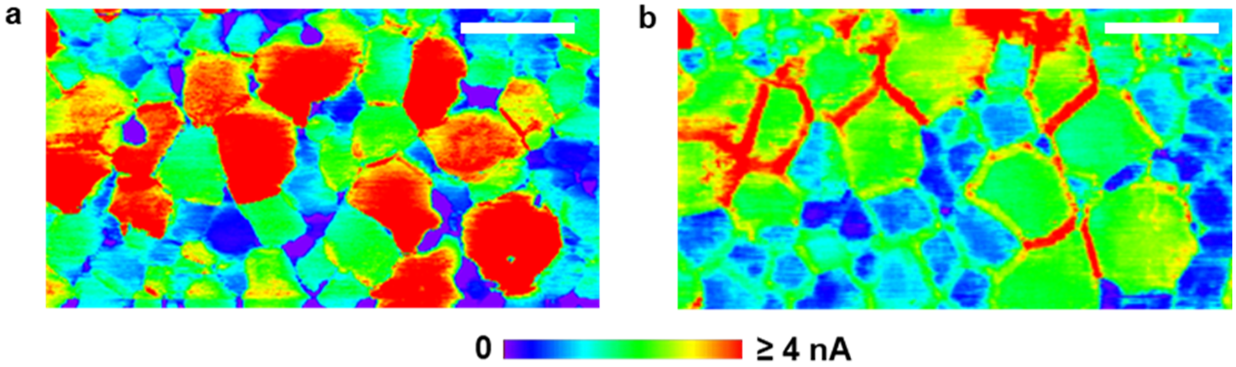

Supplementary Figure 6 | Relative photocurrent inversion. a-b, Plan-views of photocurrent mapping of as-received $MAPbI_3$ thin film top surface (**a**) and after nanomachining 100 nm into the film (**b**). The scale bars are 1 μm.

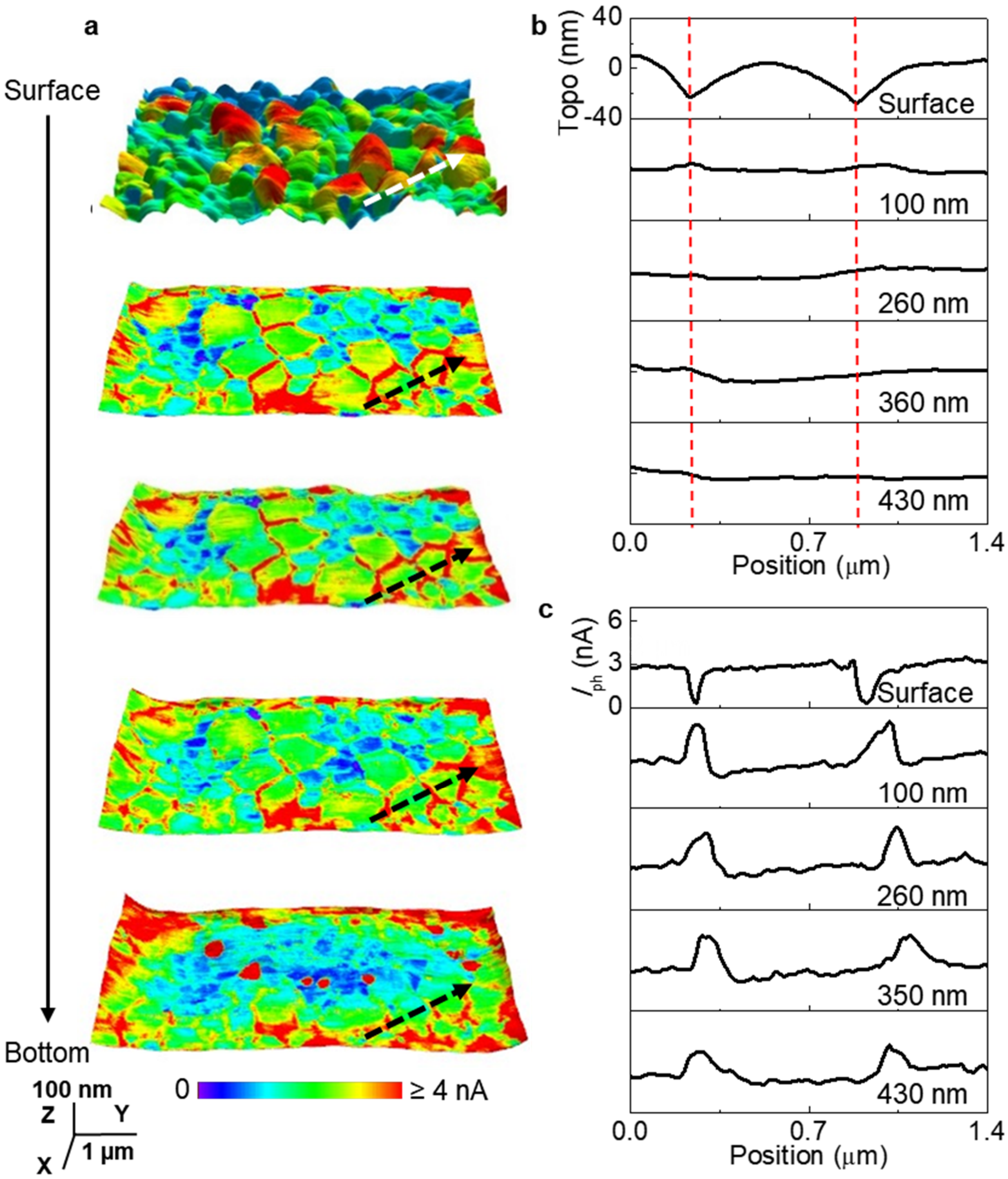

Supplementary Figure 7 | Nanomachining sequence of $MAPbI_3$ via T-AFM. a, Progressive 3D representations of the $MAPbI_3$ thin film topography overlaid with color contrast depicting the simultaneously acquired photocurrents, all for the same area, but from the as-provided top surface down to an average milled depth of 430 nm. **b**, Topography line-scans for 3 adjacent grains and 2 GBs where indicated by dashed arrows in (**a**), with the initial GB locations identified by the red dashed lines in (**b**). **c**, Equivalent photocurrent line scans across the same grain and GB locations for several depths into the film, demonstrating a uniform enhancement in the local photocurrent for GBs relative to grains beneath the surface-instead of apparently diminished GB properties at the 10× rougher, as-provided film surface.

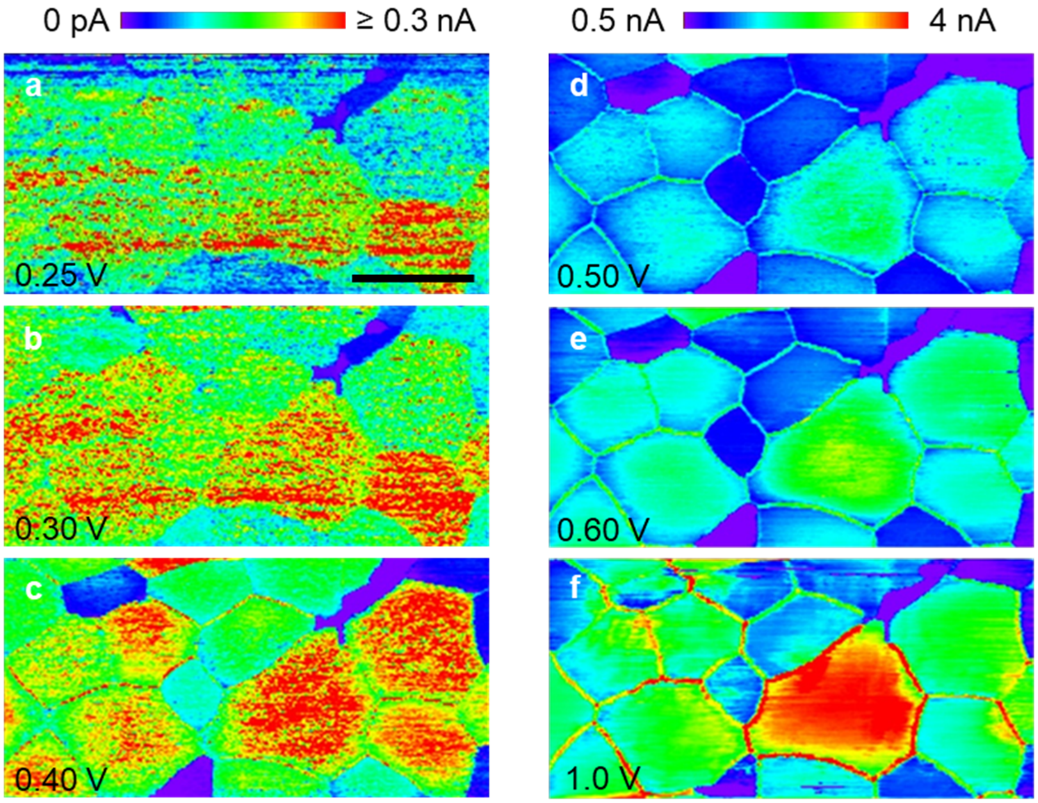

Supplementary Figure 8 | *I-V* characteristics at low bias. a-f, Montage of pc-AFM maps in a single 2 µm × 4 µm T-AFM polished region for sequentially increasing biases from 0.25 V (**a**) up to 1 V (**f**) as indicated. (**a**)-(**c**), and (**d**)-(**f**), share their own color bars as noted above (**a**) and (**d**) respectively, otherwise the low currents in (**a**) are comparatively negligible. The universal scale bar in (**a**) is 1 µm.

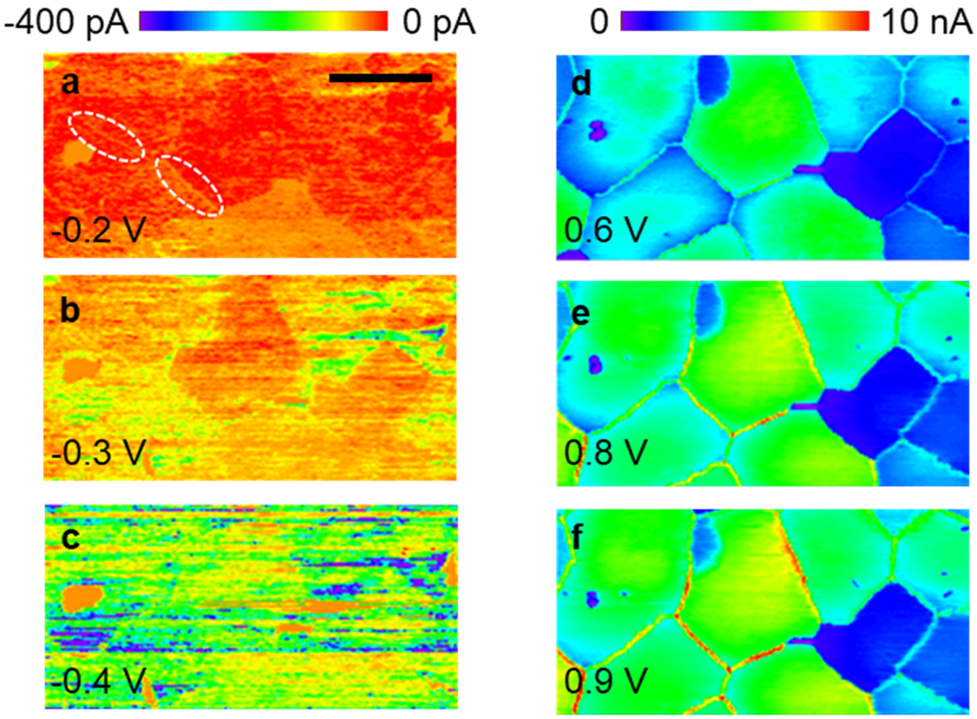

Supplementary Figure 9 | *I-V* characteristics at reverse bias. Pc-AFM maps with reverse bias in a single 2 μm × 4 μm T-AFM polished region (**a-c**) for sequentially increasing biases from -0.2 V (**a**) to -0.4 V (**c**). (**d**)-(**f**), pc-AFM maps with forward bias of 0.6 V to 0.9 V for the same region. The universal scale bar is 1 μm. Distinct contrast ranges are used for each column due to the around 25× difference and opposite polarity for the respective photocurrents.

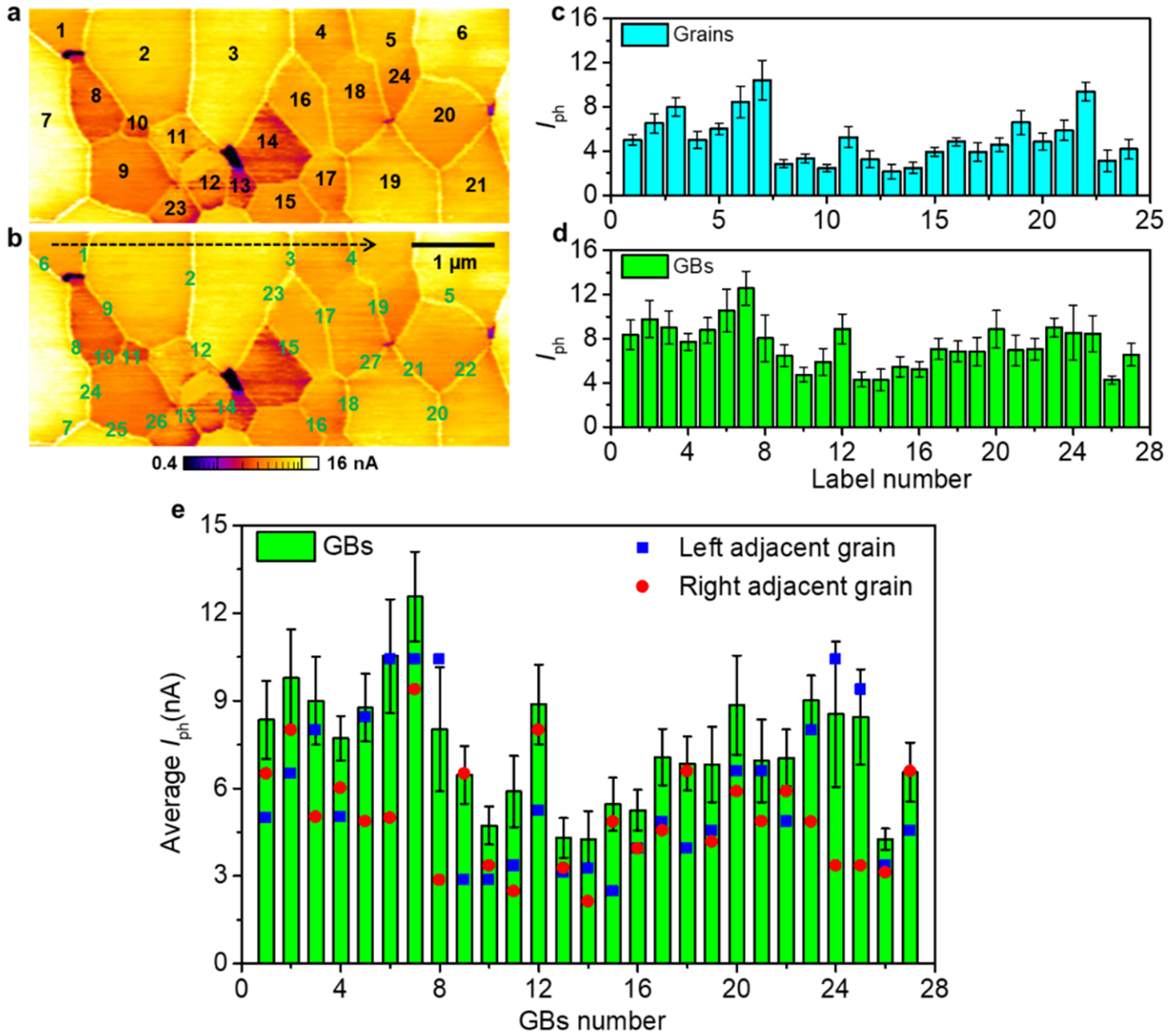

Supplementary Figure 10 | Grain-to-grain and GB-to-GB variations of I_{ph} in $MAPbI_3$ thin films. a-b, Photocurrent mapping with grain (**a**) and GB (**b**) labels, overlain on Figure 3f from the main text. **c-d**, Statistics of averaged I_{ph} levels within grains and GBs labeled in (**a**) and (**b**) respectively. **e**, Statistics of average I_{ph} on GBs and I_{ph} of the left and right adjacent grains. The Error bars show the standard deviation of the averaged I_{ph} within grains and GBs.

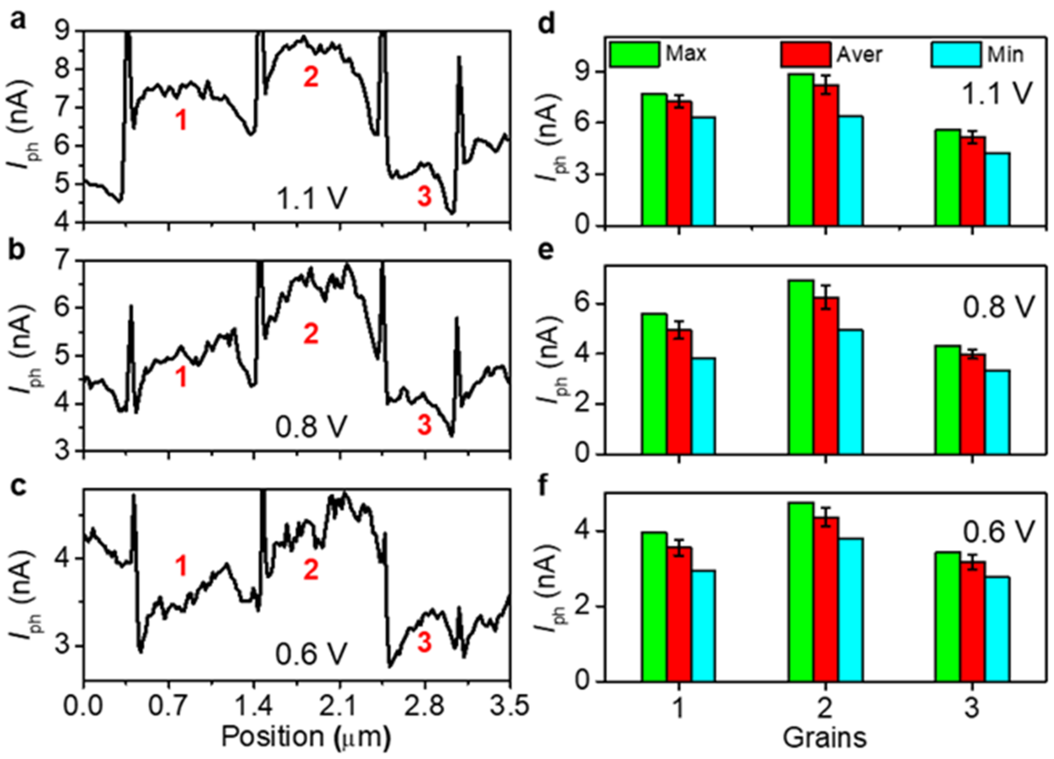

Supplementary Figure 11 | Variations of I_{ph} in single $MAPbI_3$ thin film grains. a-c. Replotted Figure 4a by zooming into I_{ph} profiles across three grains at 1.1 V, 0.8 V and 0.6 V respectively. **d-f**, Statistics of lateral I_{ph} variations of individual grains at voltages from 0.6 V to 1.1 V. The Error bars show the standard deviation of the averaged I_{ph}.

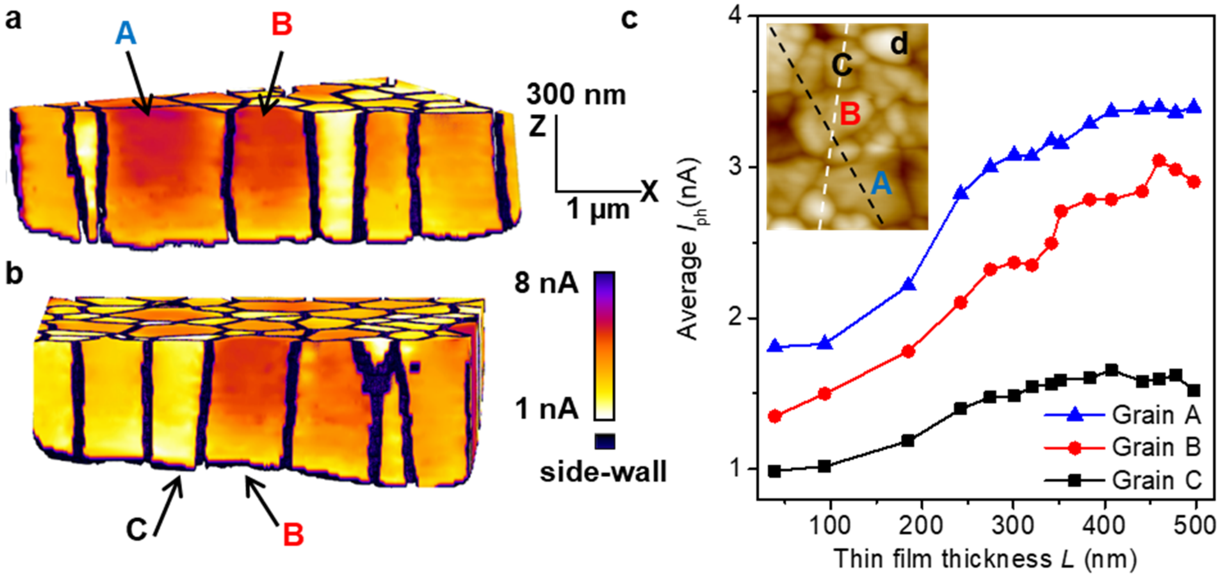

Supplementary Figure 12 | Variations of I_{ph} with depth in $MAPbI_3$ thin film grains. a-b. Cross-sectional tomogram showing evolution of I_{ph} along depths of three typical grains with different sizes and shapes marked with A, B and C, respectively. **c**, Averaged I_{ph} within three single grains along the depth of the $MAPbI_3$ thin film. **insert**, AFM topography showing the top surface morphology including the A, B and C grains, where the black and white dashed lines identify the cross-section orientations for (**a**,**b**) respectively.

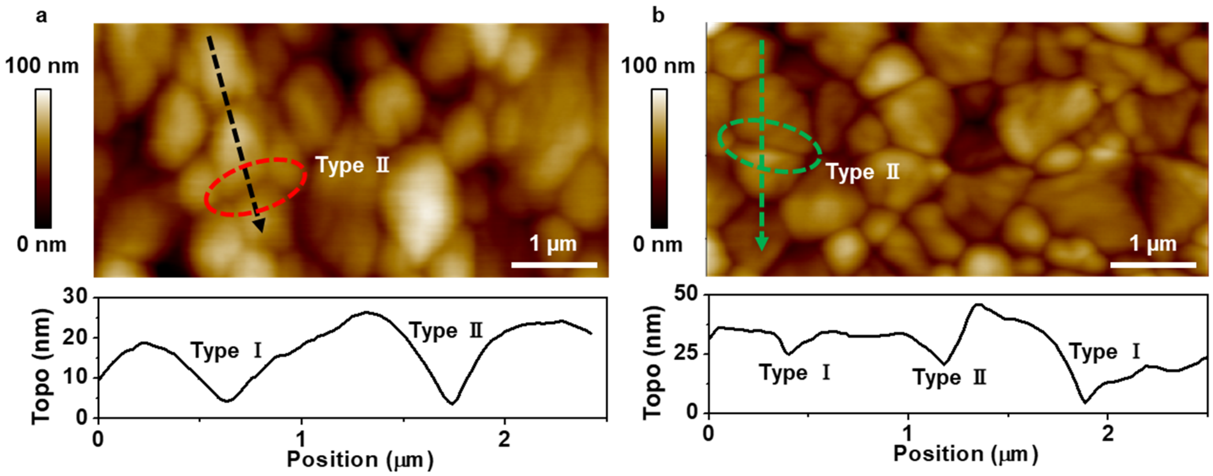

Supplementary Figure 13 | Top surface topographies of Type-I and Type-II GBs. a, Grain boundary grooves at the as-provided $MAPbI_3$ thin film surface are morphologically identical for Type-I GBs and Type-II GBs (centered within overlain ovals) and were only identified by subsequent nanomachining and voltage dependent mapping (from Figure 3). **b**, Similarly, Type-I and Type-II GBs again cannot be topographically distinguished at the initial surface until T-AFM is performed (Figure 4).

Supplementary Note 1: Nanomachined Surface During T-AFM

The nanomachining process during T-AFM gradually diminishes the surface roughness while milling into the specimen. This is exemplified by the AC-mode AFM topography image of Supplementary Figure 2a, and corresponding line-profile along the dashed arrow, revealing around 50 nm of milling and a dramatically reduced roughness. Most removed material is swept to the edges, generating sometimes substantial protrusions around the scanned periphery, while the grain boundary grooves and proud grains of the initial surface are also apparent. Zooming into the milled region where indicated by the dotted overlay, higher magnification topography (Supplementary Figure 2b) and amplitude images (Supplementary Figure 2c, especially sensitive to topographic edges) reveal that the surface becomes extraordinarily smooth in comparison to the surrounding as-provided surface. In fact, steps are reduced as much as 10× by the T-AFM milling process, to less than 2 nm, according to the displayed line-profiles.

Supplementary Note 2: Post T-AFM Surface Topography

The schematic in Figure 1a is a simplified illustration of our measurement setup, and, is not plotted with a 1:1 scale of the actual tip and sample geometry. To clarify this point, Supplementary Figure 3a shows a fully scaled cross-sectional schematic of the end of the AFM cantilever, the tip, and a typical excavated region from any given T-AFM study in the $MAPbI_3$ thin films studied herein. Supplementary Figure 3b magnifies the excavated area to especially focus on the tip and sidewall geometry. Since our $MAPbI_3$ film is relatively thin (around 560 nm), the scan area is relatively large (6 µm × 6 µm), and the tip height relatively long (approximately 10 µm), it is geometrically impossible that the back of the cantilever and the unpolished regions will be contact. It is true, of course, that such circumstances could arise for much deeper trenches, or shorter tips, unless a correspondingly larger area is nanomachined. Supplementary Figures 3c-e display the surface topography and corresponding line profiles following a complete T-AFM experiment through the entire 560 nm thick film. Similar to Supplementary Figure 2, the reductive nanomachining approach is obvious, as is the redeposition of removed material around the trenched area. These results, along with the T-AFM reconstructions in Figure 1 and Figure 4, reveal that the full excavated volume is often not an ideal cuboid. The transient region along the edges in Supplementary Figure 3d-e could be due to a combination of factors, including variations in tip-

sample interactions (forces) while climbing or descending the evolving edges, the non-uniform tip geometry (a tilted pyramid), debris pileup at edges, etc.

Supplementary Note 3: Tomographic Interpolation Distances

Although the mean depth for progressive milling steps during T-AFM is relatively uniform, locally it is not intrinsically linear. After lateral drift correction based on topographic features that are persistent over multiple consecutive imaging frames, T-AFM is therefore based on gridded data in the *xy* direction but a technically 'sparse' dataset along the z-direction. That is, pixels in the *x* and *y* axes are uniformly spaced, but may be non-uniformly spaced along *z*. For traditional 2D and 3D image analysis, the collective assembly of all 7.6 M total data points for Figure 1 are therefore interpolated into rectilinear 3-D locations via Delaunay triangulation using conventional image processing routines (Mathworks Matlab). This practically results in a distribution of interpolation distances between the initially acquired and the finally analyzed tomogram data points, as summarized in the overlain histogram (left axis) and pareto (right axis) plots of Supplementary Figure 4. For reasonably dense initial data as acquired herein, the resulting interpolated voxel positions for approximately 70% of all tomographic voxels within the milled out 'hull' are interpolated by less than the distance to the nearest adjacent acquired pixel (11.7×11.7 nm initial pixels). More than 95% of voxels are interpolated by less than 2 adjacent nearest neighbor distances (23.4 nm). Equivalently, the local milling rate during T-AFM only differed from the mean rate by more than 2 pixel dimensions for less than 5% of all acquired data. Higher z-fidelity is of course possible with more gradual milling, though practically this should only be experimentally worthwhile (it is correspondingly slower) if ultrafine variations along the *z*-direction need to be investigated.

Supplementary Note 4: Dark and Light Current Measurement During T-AFM

Supplementary Figure 5 compares pc-AFM measurements during illumination, with otherwise identical measurements but in dark conditions, confirming that our T-AFM and other pc-AFM based images are predominantly sensitive to photocarriers generated within the $MAPbI_3$ thin film. An extremely weak dark current, on the order of less than 10 pA, is typically recorded as shown in Supplementary Figure 5a at the identical location and bias as for Figure 2. Rescanning this same region during illumination, on the other hand, yields photocurrents at the nA scale as well as clearly

resolved grain and GB dependent responses (Supplementary Figure 5b, a purely plane-view version of Figure 2b). In Supplementary Figure 5c, this dramatic 1000× change is demonstrated upon switching the illumination source from OFF to ON mid-scan, including a log-scale photocurrent line scan profile along the dashed arrow as indicated.

Supplementary Note 5: 2D illustration of Relative Photocurrent Inversion

In addition to the line scans of Figures 2c-d where specified on the perspective view photocurrent and topography images of Figures 2a-b, plan-view photocurrent images are also shown here as Supplementary Figures 6a-b to further illustrate the photocurrent inversion at GBs.

Supplementary Note 6: Benefits of $MAPbI_3$ Nanomachining During T-AFM

Expanding on Figure 2a-d, Supplementary Figure 7 displays additional data comparing the topography and photocurrent of the $MAPbI_3$ specimen in the as-received state as well as during several T-AFM milling steps into the depth of the film as indicated. Once the initial surface microstructure, proud grains, and grain boundary grooves are effectively polished via T-AFM, the now enhanced photocurrent observed at GBs remains qualitatively and quantitatively consistent throughout the film thickness as far as 430 nm beneath the initial surface.

Supplementary Note 7: 2D mapping of *I-V* Characteristics at Low Bias

As pointed out in Methods, because our $MAPbI_3$ thin film is directly prepared on a clean transparent conductive electrode (FTO-glass), there is no appreciable built-in field to separate the photo-generated carriers as with a fully assembled p-n or p-i-n heterojunction solar cell. Accordingly, pc-AFM measurements on T-AFM polished regions at voltages near short circuit conditions (in fact for any bias smaller than 0.4 V) yield no clear image of distinguishable grains and GBs with sufficient signal-noise ratio to be meaningful (Supplementary Figures 8a-c). A DC bias above 0.5 V or higher, on the other hand, yields high fidelity and high signal-to-noise ratio photocurrent maps (Supplementary Figures 8d-f). More generally (for any given area we studied), we found 0.6 V to be a reasonable low-V threshold in order to get consistently high fidelity across all our datasets. Accordingly, as noted about Figure 3, we implemented bias conditions from 0.6 V up to 1.1 V, and we did not use higher voltages as we have previously observed this to potentially degrade the conductive-coating on the tip, leading to inconsistent and poor imaging quality.

Supplementary Note 8: 2D mapping of *I-V* Characteristics at Reverse Bias

For reverse bias conditions, as is shown in Supplementary Figures 9a-c, pc-AFM measurements at low reverse biases (smaller than -0.4 V) mostly show small photocurrents with poorly resolved features—similar to equally small positive bias conditions. There are occasionally some regions showing higher photocurrent on certain grains or GBs, as marked with white ovals in Supplementary Figure 9a. Applying even stronger reverse biases, such as -0.4 V and beyond, only yields distorted photocurrent signals and correspondingly damage to the $MAPbI_3$ thin film surface. For comparison, the photocurrent during positive biasing (Supplementary Figures 9d-f) was initially acquired in the same area as that studied with reverse biases in Supplementary Figures 9a-c, confirming the extremely high signal-to-noise that is possible for the 'normal' forward bias conditions equivalent to those employed in Fig 3.

Supplementary Note 9: Spatial Variations of Photocurrents in $MAPbI_3$ Thin Films

In addition to our most convincing finding of consistently higher photocurrents measured at GBs throughout the thickness of HP films, we also observe different photocurrents for distinct grains, I_{ph} variations within grains, and possibly even variations along individual grain boundaries. These results are all compatible with spatial variations of the photoresponse reported by others at the surfaces of halide perovskite semiconductors[1-4]. Even so, substantial further data comparing grain and adjacent grain boundary photocurrents, as well as variations across single grains, is available because of the novel T-AFM approach, as summarized below in Supplementary Figures 10-12, respectively.

First, Supplementary Figures 10a-b replicate the pc-AFM image from Figure 3f, but with notations overlaid to identify every individual grain (a) and GB (b) in the field of view, respectively. In addition, Supplementary Figures 10c-d includes present bar graphs of the mean photocurrents for all individual grains and GBs, where standard deviation error bars based on the photocurrents for the pixels encompassed by each distinct feature. Both grain-to-grain as well as GB-to-GB variations in photocurrents are clearly revealed. As has been pointed out from previous studies and topical reviews, this variation could be due to multiple factors, including microstructure, self-doping, and photo-response heterogeneities among different grains[1-7]. Finally, in Supplementary Figure 10e the GB data from Supplementary Figure 10d is replotted, but the mean I_{ph} signals from

the nearest neighbor grains from are overlaid based on Supplementary Figure 10c. In spite of the variations in I_{ph} among different grains, the I_{ph} at GBs is consistently higher than their immediate adjacent grains for almost every case (GBs No. 8, 24, and 25 are the only outliers).

Analyzing individual grain boundaries reveals that they also behave distinctly, likely due to different local bonding configurations, composition, and defect densities. Site-selective nanomachining during T-AFM may be crucial to investigations into such phenomena in the future, even including the possibility of 3-D variations along single grain boundaries, especially as it can literally remove any surface effects that might contribute to apparent local variations in properties (surface damage, certain exposed facets, heterogeneities from processing whether intentional or not, regions of high curvature such as grain boundary grooves as already demonstrated with Figure 1, etc.). Already from the pc-AFM results, clear lateral variations of photocurrent exist across a single grain. Supplementary Figures 11a-c replots Figure 5c by zooming into the I_{ph} profiles for three grains at three different voltages. From the I_{ph} profiles, lateral variations of photocurrent along the horizontal scan direction are clearly revealed. The statistics of the maximum, minimum and averaged I_{ph} from the line scan profiles of individual grains in Supplementary Figures 11d-f indicates approximately 20% to 45% lateral variations in I_{ph} among these representative grains.

Finally, in Supplementary Figure 12, variations of photocurrent with respect to depth are extracted from the T-AFM measurement of Figure 1. Three typical single grains are considered, marked with (A), (B) and (C) as indicated in Supplementary Figures 12 a-b. Supplementary Figure 12c reveals around 30% to 50% decrease of I_{ph} as the depth approaches the last 100 nm of film thickness. As we have described in the main text with respect to the *I-V* behavior of grains and GBs, the I_{ph} is dominated by the photogenerated carrier density (N_{ph}). In terms of film thickness (L), $N_{ph} \propto (1- \exp(-\alpha L))/L$, where α is the absorption coefficient. Therefore, a decrease in I_{ph} is expected due to the volume of absorbing $MAPbI_3$ beneath the biased probe progressively diminishing during T-AFM, with a corresponding decrease in the quantality of photogenerated carriers[8]. Theoretical modeling[9] may be helpful in the future to analytically solve the evolution of I_{ph} with respect to depth. Such depth-dependent insights are absolutely unique to T-AFM[10] and represent one of the most promising next steps for HPs investigations employing the T-AFM approach.

Supplementary Note 10: Indistinguishable Topography of Type-I and Type-II GBs at the Initial Surface

As illustrated in Supplementary Figure 13, the two GB types identified via T-AFM are indistinguishable based purely on the as-grown topography. According to the image and line profile across the as-provided surface in Supplementary Figure 13a, there is no apparent morphological difference between the grain boundary grooves at Type-I GBs compared to Type II GBs. These are only ultimately distinguished via voltage dependent investigations as in Figure 3. The Type II GB (dashed oval) at the as-provided surface of Supplementary Figure 13b is similarly impossible to topographically differentiate from the more common surrounding Type I GBs, only becoming apparent upon subsequent T-AFM imaging (Figure 4e).

Supplementary References

1. Son, D.-Y. *et al.* Self-formed grain boundary healing layer for highly efficient $CH_3NH_3PbI_3$ perovskite solar cells. *Nat. Energy* **1**, 16081(2016).
2. Jiang, Q. *et al.* Surface passivation of perovskite film for efficient solar cells. *Nat. Photonics* **13**, 460-466 (2019).
3. Adhyaksa, G. W. P. *et al.* Understanding Detrimental and Beneficial Grain Boundary Effects in Halide Perovskites. *Adv. Mater.* **30**, 1804792 (2018).
4. Tennyson, E. M., Doherty, T. A. S. & Stranks, S. D. Heterogeneity at multiple length scales in halide perovskite semiconductors. *Nat. Rev. Mater.* **4**, 573-587 (2019).
5. Wang, Q. *et al.* Qualifying composition dependent p and n self-doping in $CH_3NH_3PbI_3$. *Appl. Phys. Lett.* **105**, 163508 (2014).
6. Yin, W.-J., Shi, T. & Yan, Y. Unusual defect physics in $CH_3NH_3PbI_3$ perovskite solar cell absorber. *Appl. Phys. Lett.* **104**, 063903 (2014).
7. Stavrakas, C. *et al.* Probing buried recombination pathways in perovskite structures using 3D photoluminescence tomography. *Energ Environ. Sci.* **11**, 2846-2852 (2018).
8. Nelson, J. *The Physics of Solar Cells*, 88-89 (Imperial College Press 2003).
9. Reid, O. G., Munechika, K. & Ginger, D. S. Space Charge Limited Current Measurements on Conjugated Polymer Films using Conductive Atomic Force Microscopy. *Nano Lett.* **8**, 1602-1609 (2008).
10. Steffes, J. J., Ristau, R. A., Ramesh, R. & Huey, B. D. Thickness scaling of ferroelectricity in $BiFeO_3$ by tomographic atomic force microscopy. *Proc. Natl. Acad. Sci.* **116**, 2413-2418 (2019).
