# Supplementary material for: Anomalous 3D nanoscale photoconduction in hybrid perovskite semiconductors revealed by tomographic atomic force microscopy
Source: Nat Commun. 2020 Jul 3;11:3308. doi: 10.1038/s41467-020-17012-y (PMC7335063; doi:10.1038/s41467-020-17012-y)
Supplement: Supplementary file 2 — Description of Additional Supplementary Files [file 41467_2020_17012_MOESM2_ESM.pdf]

Description of Additional Supplementary Files

Supplementary Movie 1

3D consecutive cross-sectional views of the photocurrent tomogram of the polycrystalline $MAPbI_3$ thin film.

Supplementary Movie 2

Segmented 3D consecutive cross-sectional views of the photocurrent distribution along GBs of the polycrystalline $MAPbI_3$ thin film.

Supplementary Movie 3

Segmented 3D consecutive cross-sectional views of photocurrent distribution in grains of the polycrystalline $MAPbI_3$ thin film.
